# Supplementary material for: Causal Relationship between Aging and Anorexia Nervosa: A White-Matter-Microstructure-Mediated Mendelian Randomization Analysis
Source: Biomedicines. 2024 Aug 16;12(8):1874. doi: 10.3390/biomedicines12081874 (PMC11351342; doi:10.3390/biomedicines12081874)
Supplement: Supplementary file 1 [file biomedicines-12-01874-s001.zip › Supplementary Figure/Figure S4/Singe SNP plot of MR analysis for TL to ubm-b-1785.pdf]

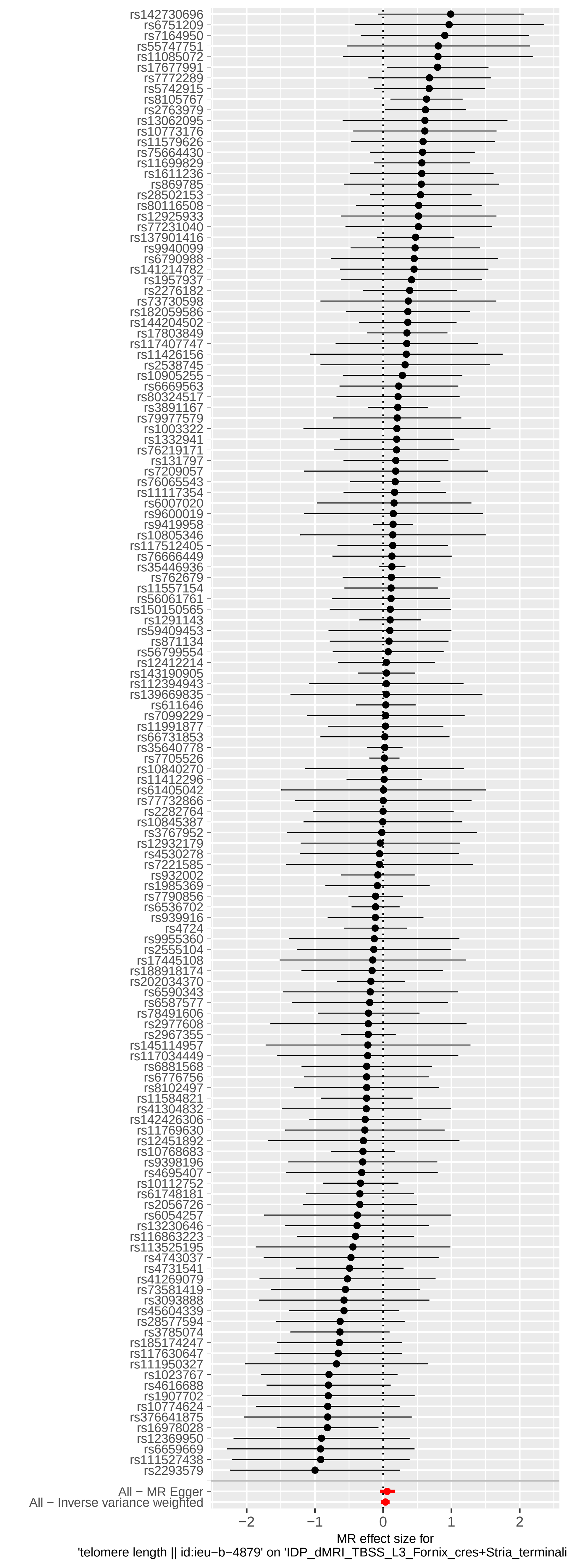
rs142730696
rs6751209
rs7164950
rs55747751
rs11085072
rs17677991
rs7772289
rs5742915
rs8105767
rs2763979
rs13062095
rs10773176
rs11579626
rs75664430
rs11699829
rs1611236
rs869785
rs28502153
rs80116508
rs12925933
rs77231040
rs137901416
rs9940099
rs6790988
rs141214782
rs1957937
rs2276182
rs73730598
rs182059586
rs144204502
rs17803849
rs117407747
rs11426156
rs2538745
rs10905255
rs6669563
rs80324517
rs3891167
rs79977579
rs1003322
rs1332941
rs76219171
rs131797
rs7209057
rs76065543
rs11117354
rs6007020
rs9600019
rs9419958
rs10805346
rs117512405
rs76666449
rs35446936
rs762679
rs11557154
rs56061761
rs150150565
rs1291143
rs59409453
rs871134
rs56799554
rs12412214
rs143190905
rs112394943
rs139669835
rs611646
rs7099229
rs11991877
rs66731853
rs35640778
rs7705526
rs10840270
rs11412296
rs61405042
rs77732866
rs2282764
rs10845387
rs3767952
rs12932179
rs4530278
rs7221585
rs932002
rs1985369
rs7790856
rs6536702
rs939916
rs4724
rs9955360
rs2555104
rs17445108
rs188918174
rs202034370
rs6590343
rs6587577
rs78491606
rs2977608
rs2967355
rs145114957
rs117034449
rs6881568
rs6776756
rs8102497
rs11584821
rs41304832
rs142426306
rs11769630
rs12451892
rs10768683
rs9398196
rs4695407
rs10112752
rs61748181
rs2056726
rs6054257
rs13230646
rs116863223
rs113525195
rs4743037
rs4731541
rs41269079
rs73581419
rs3093888
rs45604339
rs28577594
rs3785074
rs185174247
rs117630647
rs111950327
rs1023767
rs4616688
rs1907702
rs10774624
rs376641875
rs16978028
rs12369950
rs6659669
rs111527438
rs2293579
All − MR Egger
All − Inverse variance weighted
−2
−1
0
1
2
MR effect size for
'telomere length || id:ieu−b−4879' on 'IDP_dMRI_TBSS_L3_Fornix_cres+Stria_terminali
